# Extreme river flood exposes latent erosion risk

**DOI:** 10.1038/s41586-025-09305-3

**Published:** 2025-07-10

**Authors:** H. J. Barneveld, R. M. Frings, E. Mosselman, J. G. Venditti, M. G. Kleinhans, A. Blom, R. M. J. Schielen, W. H. J. Toonen, D. Meijer, A. J. Paarlberg, R. P. van Denderen, J. S. de Jong, J. G. W. Beemster, L. A. Melsen, A. J. F. Hoitink

**Affiliations:** 1 Hydrology and Environmental Hydraulics Group, Department of Environmental Sciences, Wageningen University and Research, Wageningen, the Netherlands; 2 HKV, Lelystad, the Netherlands; 3 DG Rijkswaterstaat, Ministry of Infrastructure and Water Management, Utrecht, the Netherlands; 4 Deltares, Delft, the Netherlands; 5 Faculty of Civil Engineering and Geosciences, Delft University of Technology, Delft, the Netherlands; 6 School of Environmental Science, Simon Fraser University, Burnaby, British Columbia Canada; 7 Department of Physical Geography, Utrecht University, Utrecht, the Netherlands; 8 Department of Earth Science, Vrije Universiteit Amsterdam, Amsterdam, the Netherlands; 9 RiQuest, Nijkerkerveen, the Netherlands; 10 Present Address: Sweco Sverige, Stockholm, Sweden

**Keywords:** Natural hazards, Environmental impact, Geomorphology

## Abstract

Climate change is expected to increase the frequency and magnitude of river floods^1^. Floods not only cause damage by inundation and loss of life^2,3^ but also jeopardize infrastructure because of bank failure and riverbed erosion processes that are poorly understood. Common flood safety programmes include dyke reinforcement and river widening^4–9^. The 2021 flood in the Meuse Basin caused 43 fatalities and billions of dollars of damage to infrastructure^10^. Here, on the basis of analysis of the Meuse flood, we show how uneven widening of the river and heterogeneity of sediment deposits under the river can cause massive erosion. A recent flood safety programme widened the river^11^, but created bottlenecks where widening was either prevented by infrastructure or not yet implemented. Riverbed erosion was exacerbated by tectonic uplift that had produced a thin top gravel layer above fine-grained sediment. Greatly enhanced flow velocities produced underwater dunes with troughs that broke through the gravel armour in the bottlenecks, exposing easily erodible sands, resulting in extreme scour holes, one more than 15 m deep. Our investigation highlights the challenges of re-engineering rivers in the face of climate change, increased flood risks and competition for river widening space, and calls for a better understanding of the subsurface.

## Abstract (web summary)

Analysis of the 2021 Meuse River flood reveals potential erosion risks due to uneven river widening and subsoil variability, highlighting challenges for river engineering and flood mitigation in a changing climate.

## Main

Humans have increasingly altered the course and layout of rivers, reducing the number of large free-flowing rivers by 63% (ref. ^12^) and causing widespread channel narrowing and incision^13–15^. Channel incision lowers water levels, whereas narrower floodplains due to increased human activity along rivers raise flood levels^16^. Meanwhile, climate change increases the frequency and magnitude of extreme weather events, and negatively affects ecosystem services such as water availability, biodiversity, navigation and flood safety^17^. Riverbed erosion and deposition affect all of these services. Slow, long-term erosion trends are well documented for many rivers, including the Danube^18^, the Mississippi^19^, the Rhine^15,20–24^, various Italian rivers^25^ and the Yangtze^26^.

Numerical models are widely used to predict future riverbed changes and to assess climate change impacts^27,28^. Although these models can anticipate gradual trends in riverbed evolution under average hydrological conditions, they fail to accurately predict the location and scale of morphodynamic processes during extreme floods. Flood peaks can elicit major changes, such as avulsion^29^ and extreme erosion and deposition at the riverbed^30^, banks^31^ and floodplains, which damage infrastructure and affect flood safety^32^. Rapid morphodynamic processes during floods are poorly understood^23^ and often ignored in long-term flood level predictions.

The 2021 Meuse River flood offered a unique opportunity to study how extreme floods alter river morphology. Erosion, deposition and flow conditions were monitored before, during and after the flood event, and detailed geological data on the subsurface are available. We document massive erosion and deposition, and identify the main triggers and processes. Our findings expose the potential erosion risks faced by engineered rivers in heterogeneous subsoils worldwide, for which climate change demands new flood mitigation strategies.

## River Meuse morphology and flood history

**Extended Data Fig. 1:**
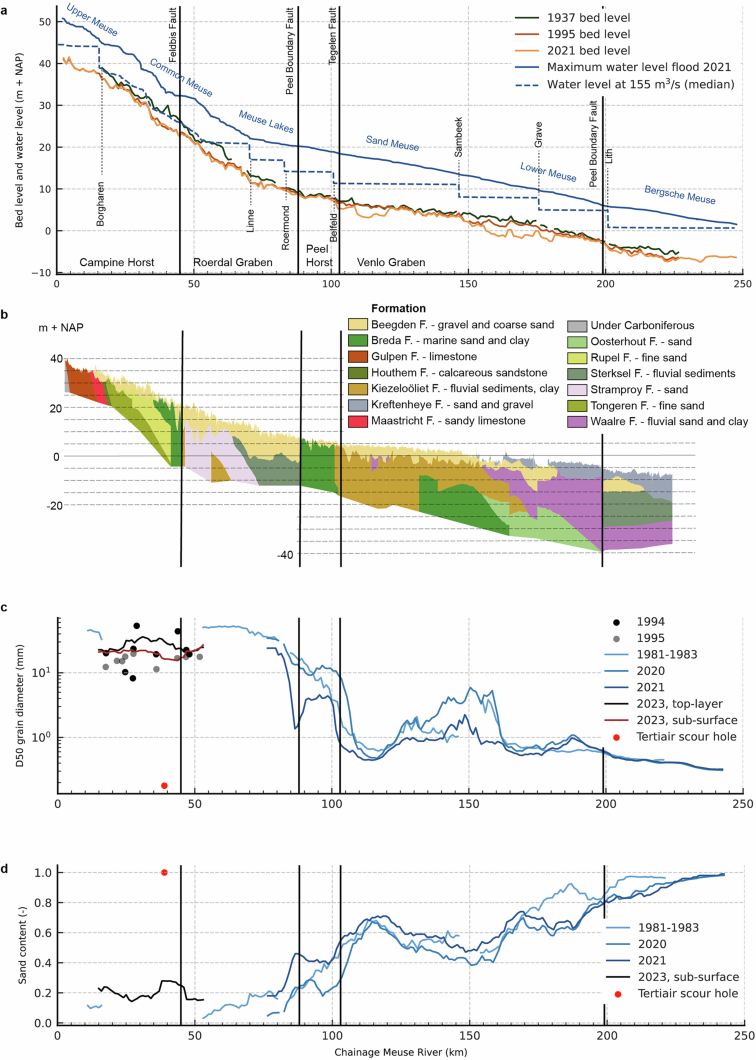
Geology, bed profile development and riverbed composition of the Meuse River. **a**, Longitudinal bed level profiles in 1937, 1995 and 2021 averaged over 1 km (yearly survey Rijkswaterstaat), peak water level profile July 2021 (Rijkswaterstaat) and locations of faults (vertical lines), graben and blocks^75^. The riverbed incises due to barrages, narrowing, bank protection, bend cut-offs and sediment extraction. **b**, Geological profile based on drillings in banks and floodplains, indicating gravel in yellow^76^. The Campine Horst rises approximately 6 mm/100 yr^34^, relative to the slowly subsiding Roerdal Graben, explaining that, over millions of years, the incision thinned the gravel layer in the Campine Horst. The green colours underneath the gravel layer in the reach km 20-40 indicate fine marine sands from the Miocene (23-5 million B.P.), Rupelian (34-28 million years B.P.) or Late Eocene/Early Oligocene (37-30 million years B.P.). **c**, Characteristic grain size diameter *D*_*50*_ from riverbed top layer (10 cm) and development in time (data Rijkswaterstaat). Data from 1981–1983, 2020 and 2021 (after the flood) are based on samples every 500 m; lines show a moving average over 10 km. **d**, Sand content top-layer in time (data Rijkswaterstaat), as a moving average over 10 km. The composition and location of the gravel-sand transition in the reach km 90–105 remained stable over the past 50 years. In parts of the Meuse River with a gravel bed, an armour layer on the riverbed is formed outside periods of high flows.

The River Meuse basin covers about 33,000 km^2^. This rain-fed, fast-responding river is almost 950 km long, of which 320 km is in the Netherlands^33^. The first 80 km of the Dutch Meuse River has a slope of approximately 0.5 m per kilometre. For 46 km in this reach, it constitutes the border between Belgium and the Netherlands, and is known as the Common Meuse. Here, it crosses the horst–graben blocks of the Roer Valley Rift System^34^, where horst uplift has thinned a gravel layer that covers fine marine sediment (Extended Data Fig. 1b). In the gravel layer, a coarse armour layer on the riverbed surface protects finer-grained subsurface sediments from erosion during normal flow conditions. During exceptionally high flood flows, this armour layer can be eroded, exposing the finer sediment below^35^. Human interference over the past 200 years included the construction of more than 70 barrages, channel shortening by bend cutoffs, dredging and sediment mining. This has led to incision of the Common Meuse up to 2 cm yr^−1^, slowing to 0.6 cm yr^−1^ in the period 1995–2017 (ref. ^36^). The largest floods since 1911, when daily monitoring started, occurred in 1926, 1993 and 1995, all during winter. After the 1995 flood, the Meuse Program was implemented to improve flood safety, including construction and strengthening of dykes, and river widening^11^.

## The 2021 summer flood in the Meuse Basin

**Fig. 1:**
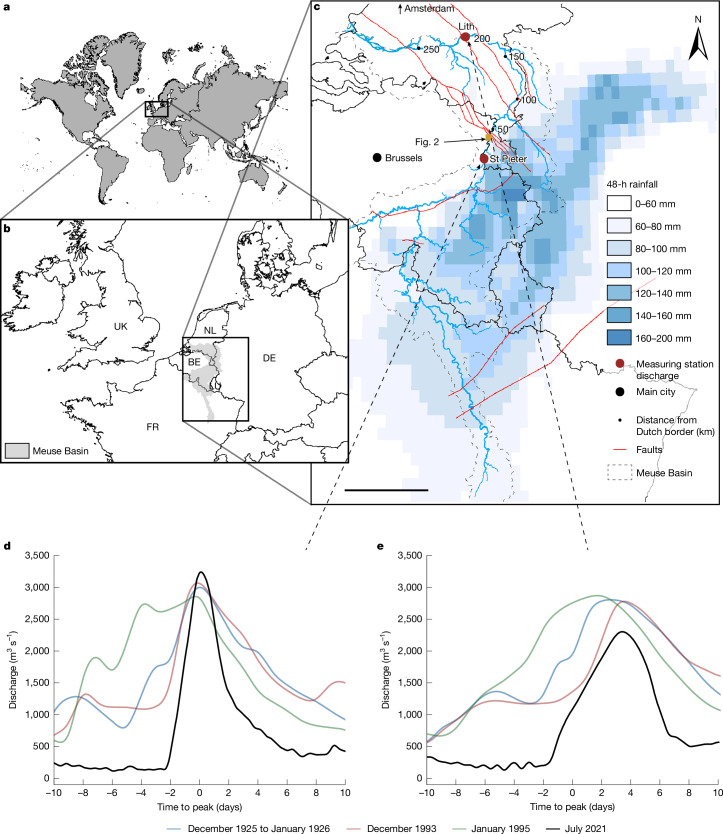
The extreme 2021 summer flood in the Meuse Basin. **a**, Location of Western Europe. **b**, Location of Meuse Basin. BE, Belgium; DE, Germany; FR, France; NL, the Netherlands. **c**, Meuse Basin with the cumulative 48-h rainfall on 13–14 July 2021 in millimetres, locally assessed as a 1,000-year event^37^. Numbers in the map show river kilometres from the Dutch border. Scale bar, 80 km. **d**, Observed discharges at St Pieter, Maastricht, km 10.8, for floods in 1926, 1993, 1995 and 2021. **e**, Observed discharges at Lith, km 202.4, according to the moment of the peak at St Pieter, showing larger wave damping for the 2021 flood. Geographical details of the Dutch Meuse River, such as slope, incision trend and sediment composition, are presented in Extended Data Fig. 1a–d. Source data

In July 2021, the stationary, cold-core low-pressure system Bernd developed over Western Europe, accompanied by an upper-level cold pool of air, attracting humid air^37,38^ and causing extreme precipitation (Fig. 1a). A World Weather Attribution study^37^ found this event to be consistent with the extremes expected in a warming climate, with human-induced climate change almost certainly increasing its likelihood and intensity. The resulting floods in tributaries of the rivers Rhine and Meuse killed approximately 240 people and caused more than US $43 billion in damage^10^.

Within 2 days, the Meuse River discharge near Maastricht (the Netherlands) increased from less than 50 m^3^ s^−1^ to a peak of 3,310 m^3^ s^−1^. The peak of the approximately 100-year flood^39,40^ exceeded the three highest discharge peaks of the past century (Fig. 1d). In the steep Common Meuse, the flood wave travelled fast and relatively undistorted. Downstream, the more gentle, wider river valley and multiple large lakes damped and delayed the sharp-peaked 2021 flood more than the broader historical floods (Fig. 1d,e), conveying it as a 10- to 15-year flood to the downstream reach.

## Impacts

**Fig. 2:**
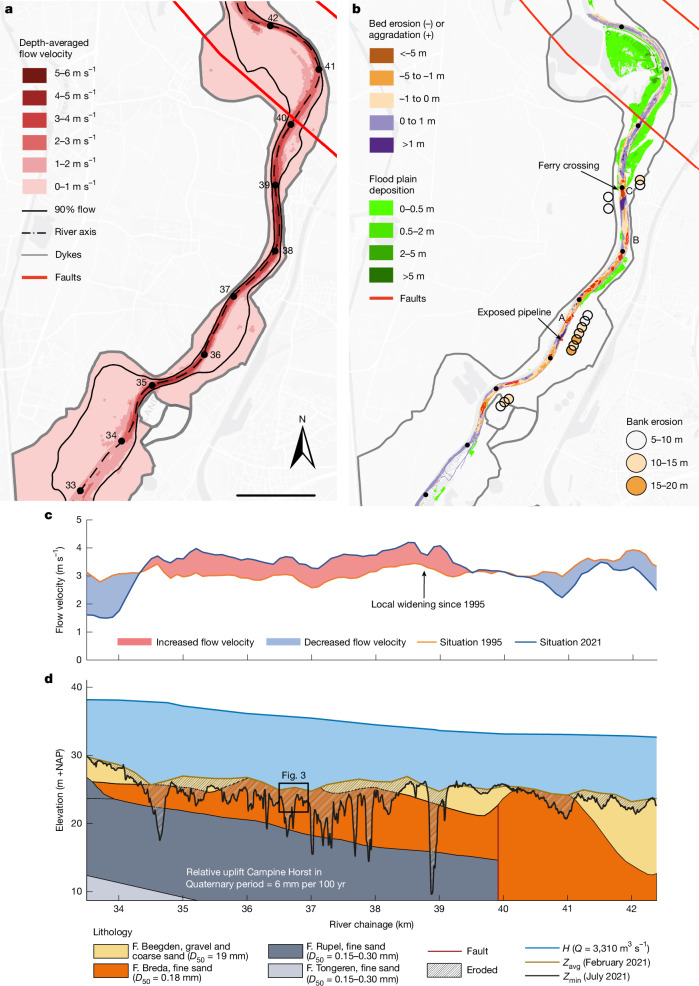
Simulated flow conditions and observed erosion in the river reach with deep scours during the 2021 flood. **a**, Simulated peak flow velocities and bounds of the area where 90% of the flow passes, revealing bottlenecks between km 35 and 39 (location indicated in Fig. 1c) due to uneven widening (Extended Data Fig. 2c). Scale bar, 1.3 km. **b**, Scour holes (red contours), bank erosion and floodplain deposits in the Common Meuse. Bed and bank scour appear to alternate (for example, along km 34.5–35.5, 36–37 and 38.5–39.2). Locations A–C refer to Fig. 3. Extended Data Figs. 4–6 provide erosion and deposition volumes and composition for the entire Dutch Meuse. **c**, Simulated maximum flow velocities and differences in the main channel, averaged over depth and 50 m of width, for the river geometries in 1995 and 2021, showing acceleration in the bottleneck section (km 35–39), and velocity reduction in widened sections (upstream km 34, at km 38.7 and downstream km 40). **d**, Bed levels (*Z*), erosion and water levels (*H*) at the peak discharge (*Q*) of 3.310 m^3^ s^−1^ in the reach of the Campine Horst. Levels relative to the Dutch reference level NAP. The deepest points (thalweg) in July 2021 are compared to the cross-section-averaged bed levels every 250 m in February 2021. Lithology with formations (F.) from Extended Data Fig. 2b. Despite the thin gravel layer at km 40–41, erosion is limited owing to reduced flow velocities in the widened section. *D*_50_, median grain diameter. Source data

During the flood, flow velocities locally exceeded 5 m s^−1^ (Fig. 2a). Narrow sections experienced substantial riverbank erosion and riverbed deepening, damaging ferry landings and exposing crucial pipelines^39^. Chemical transport through the pipelines was halted, and extensive emergency repairs had to be carried out.

A bed level survey 3 days after the flood peak revealed 16 deep scour holes, 1 more than 15 m deep, in a 6-km-long, narrow section of the Common Meuse (Fig. 2b,d). The observed alternation of bank and riverbed erosion in Fig. 2b suggests that the weakest of those two determines which is more likely to erode. At the chemical pipeline, crossing near km 36.3, the riverbed was protected to prevent vertical erosion. However, the bank eroded laterally over more than 30 m, beyond the limits of the bed protection, and then scoured vertically, exposing the buried pipeline.

The depths of the scour holes locally reached 200% of the flow depth, and were similar to scours in other rivers^41,42^. Nearly 500,000 m^3^ of sediment was eroded from the riverbed (90%) and banks (10%) of the Common Meuse, exceeding the estimated average annual flux of sand and gravel tenfold (ref. ^43^). Fine-grained marine sands of Neogene age eroded from the scour holes. Fieldwork and laser altimetry (LiDAR) showed that 50% of this material was deposited on the floodplains and in lakes within 5 km of the last scour hole, locally over 3 m thick (Fig. 2b). An additional 30% of the eroded sand was deposited within the next 25 km. The remaining 20% travelled downstream in suspension, deposited on the floodplains further downstream, as was substantiated by the granulometric composition and chemical properties of floodplain deposits (Extended Data Fig. 5).

**Fig. 3:**
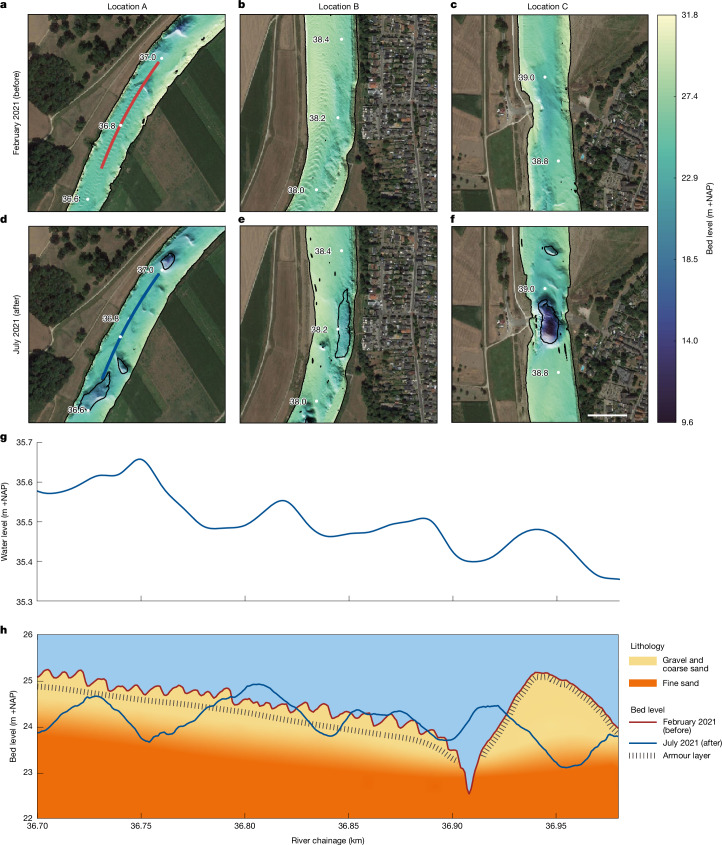
Examples of riverbed erosion during the flood. Illustrated by bed levels before (February 2021, maximum discharge 1,300 m^3^ s^−1^) and just after the July 2021 flood. **a**,**d**, Example of riverbed erosion due to dune development (location A in Fig. 2b), flow direction from bottom to top. **b**,**e**, Riverbed erosion in outer bend (km 38.2, B in Fig. 2b). **c**,**f**, Constriction scour in the narrow ferry crossing (km 38.9, C in Fig. 2b). Scale bar, 100 m. **g**, Water level profile from a LiDAR flight on 16 July, showing the presence of dunes. **h**, Detail of Fig. 2d, observed bed level and sedimentological profile along the lines in **a**,**d**, showing small (February 2021) and large (19 July 2021) dunes. The shifts in peak locations of water level (**g**) and dune troughs (**h**) are due to dune propagation between the LiDAR and riverbed surveys. The gravel and coarse sand layer thickness was estimated using the geological profile in Extended Data Fig. 1b. Source data

**Extended Data Fig. 2:**
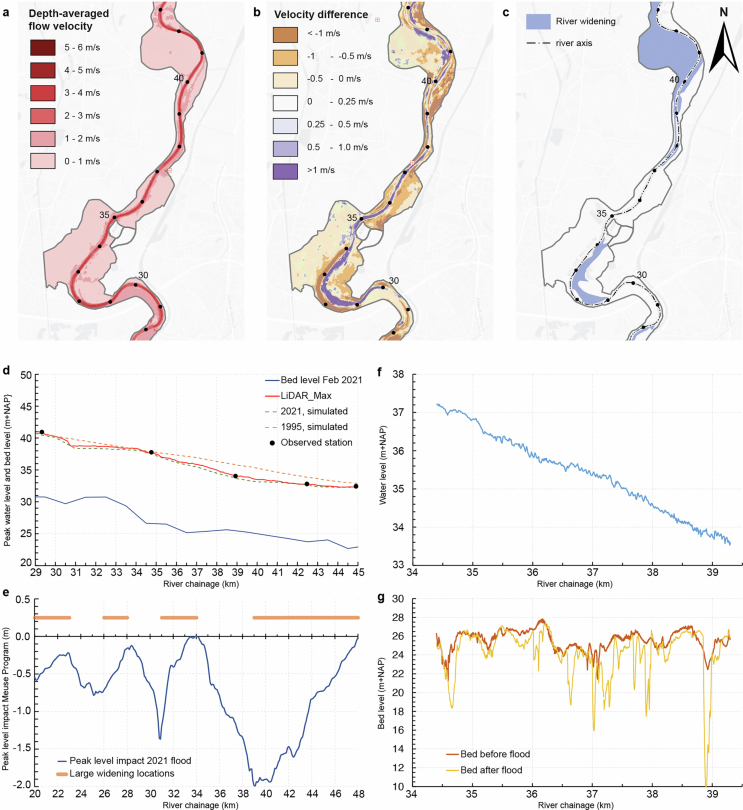
Hydrodynamic simulations and observations of the peak event. **a**, Depth-averaged maximum flow velocities in simulations with the river geometry from 1995 (before Meuse Program), obtained with the 2-dimensional Delft3D-FM model and the 2021 flood discharge as boundary condition. **b**, Difference in maximum flow velocity for the same discharge wave in simulations adopting the river geometry of 2021 before the flood, revealing much higher peak velocities in the main channel of reach km 34–39. **c**, Meuse Program measures implemented up to 2021 in reach km 34–39. **d**, Predicted (based on 2-dimensional WAQUA-simulations) and observed peak water levels at gauging stations and from the LiDAR-campaign (source Rijkswaterstaat). River widening has drawn down water levels in the narrow reach at km 34–40, increasing the flow velocities there (panel b). 2021 predicted water levels underestimate measured water levels by up to 0.5 m^39^. **e**, Impact of the Meuse Program river widening on peak water levels under 2021 discharge conditions. The flood level lowering varies strongly along the river, reducing to zero where the river was not widened over several kilometers. **f**, LiDAR based water levels along the river axis, 15 h after the peak passed. The regular water level fluctuations between the scours indicate the presence of dunes. **g**, bed level along the river axis (horizontal scale identical to panel **f**) in February 2021 (Bed before flood) and July 2021, 3 days after the flood peak. The scour holes and large dunes in between them are clearly visible after the flood.

Hydrodynamic simulations for conditions mimicking the flood event underestimated peak water levels by up to 0.5 m in the reach with the scour holes^39^. Although dense summer vegetation, crops and thick floodplain deposits may explain part of this discrepancy, large riverbed dunes may have played a role as well. LiDAR water surface observations (Fig. 3g and Extended Data Fig. 2f), taken just after the flood peak, and the post-flood bathymetry (Fig. 3d,h) indicate that dunes of 1 m or higher occurred, which is much larger than the approximately 0.2-m-high bedforms during normal floods (Fig. 3h, February 2021 bed). We calculated that energy losses due to the development of large dunes in the main channel could have raised the peak water levels by up to 0.25 m.

## Timing of scour occurrence

The geological structure surrounding the Common Meuse favours erosion in the Campine Horst where a thin gravel layer, locally less than 1 m thick, covers marine Neogene sands that, with a grain diameter of 105–210 µm, are more than 100 times finer than the gravel layer. The gravel layer is thicker (5–10 m) in the neighbouring graben block sections. This explains why scours occurred in the horst section, but not why they did develop in 2021 and did not emerge during the equally extreme 1993 and 1995 floods. Two other developments in the river explain this.

First, the gravel layer on top of the fine marine sands has thinned since 1995. Average riverbed erosion in the section of the scours was less than the current average erosion rate of 0.6 cm yr^−1^ on the Common Meuse^36^. The gravel layer nonetheless thinned by about 10 cm since 1995, because after closure of nearby coal mines, rising groundwater levels induced a regional surface uplift of on average 3.5 mm yr^−1^ since 1992 (ref. ^44^).

**Extended Data Fig. 3:**
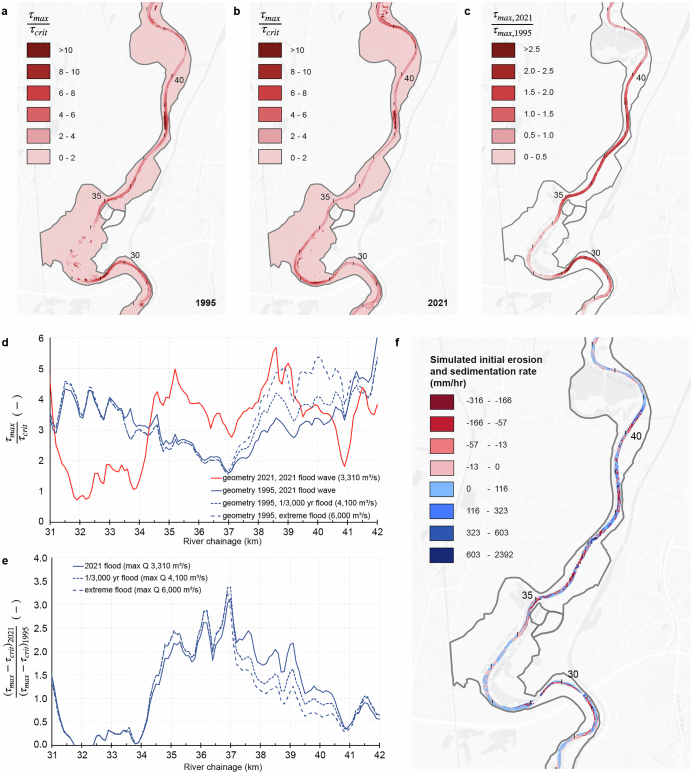
Bed shear stress variation and sediment transport capacity in 2021 and 1995. **a**, Ratio of simulated maximum to critical shear stress in the 1995 geometry (before widening) under 2021 flood conditions. **b**, Same as (a), yet adopting the 2021 river geometry, before the flood. **c**, Ratio of simulated maximum shear stress in 2021 and 1995 geometries in the main channel, adopting 2021 flood event discharge time series. **d**. Ratios of panel a. and b. projected on the river axis (average value in a 50 m circle). The ratio of maximum to critical shear stress is plotted for a 1/3000 per year flood event (peak discharge 4,100 m^3^/s) and an extreme 6,000 m^3^/s flood event, if these would have taken place in 1995. Even for more extreme flood events in the 1995 geometry, shear stresses are predominantly lower than during the 2021 flood event (T = 100 yr). **e**. Ratios of sediment transport capacity in 2021 and 1995 based on simulated bed shear stresses, adopting the Meyer-Peter and Müller (MPM) transport formula which relates sediment transport capacity to exceedance of critical shear stress to the power 1.5^77^. Values > 1 indicate that the mobility of the top sediment layer in 2021 was larger than in 1995 for similar or even more extreme flood events. The sharp increase and peaks in the sections km 34.5–35, 35.5–36, 36.5–37, 37.5–38, 38.5 and 39 correspond to the locations of the 2021 scours (Fig. 2b and d). **f**. Initial erosion and deposition rates based on a simulation with the steady peak discharge of 2021, top layer grain diameters (monitoring campaign 2023), adopting the MPM transport predictor with hiding and exposure^73^, and no morphological changes. The locations with large initial erosion (bright red) correspond well to the scour locations (Fig. 2b).

Second, the main channel of the Common Meuse has been broadened and floodplains have been excavated since 1995, lowering flood water levels. Observed water levels at discharges of 1,500–2,000 m^3^ s^−1^ dropped by up to 2 m at the ferry crossing (km 39) owing to the widening, which is reproduced in the 2021 peak flow simulation (Extended Data Fig. 2e). At locations without widening or excavation, bottlenecks in flow conveyance formed, such as at the section with scours (km 34–39). Here, water level slopes steepened, and flow strength increased. Numerical modelling shows that the maximum flow velocities in the main channel where scour occurred were up to 30% or 1 m s^−1^ higher during the 2021 flood than what they would have been in the 1995 situation under the same flood wave (Fig. 2c). In widened sections, the flow velocities decreased. We compared bed shear stresses and exceedance of sediment entrainment thresholds before and after widening, as erosion potential indicators. This revealed that sediment mobility in 2021 was up to 5.5 times higher and more variable in space than what it was for an identical flood in 1995 (Extended Data Fig. 3). The simulations further showed that even for a hypothetical flood peak of 6,000 m^3^ s^−1^ in 1995, sediment would not have become as mobile as it became in 2021, which shows the magnitude of the impact of uneven river widening. The computed bed shear stresses were sufficiently high to mobilize cobbles in the armour layer. Increased fine sand supply from upstream may have further enhanced the mobility of coarse armour sediment^35^ in 2021. Measured water levels suggest that large dunes in the bottlenecks contributed to armour breakup^45–47^ and triggered rapid, deep erosion when the dune troughs scoured into the underlying Neogene sands.

The formation of a flow conveyance bottleneck due to uneven widening was the most likely cause of the formation of deep scours, which was exacerbated by the local geologic structure, bedforms that broke through the top gravel layer, and other local factors, including natural deepening at river bend pools (Fig. 3b,e) and the main channel constriction (Fig. 3c,f).

### Implications for other rivers

**Fig. 4:**
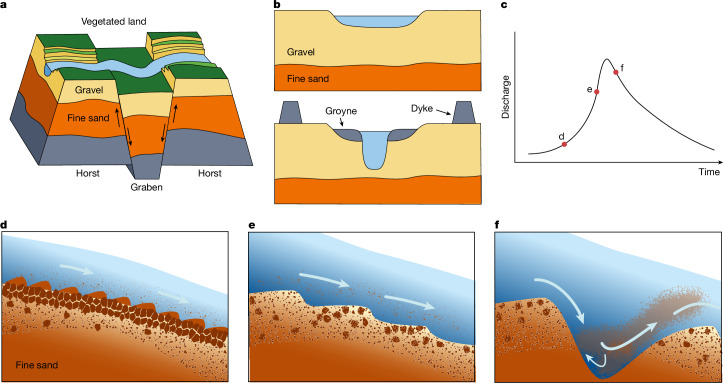
Long- and short-term processes that contribute to sudden scour hole formation. **a**, Geological timescale, with typical uplift and subsidence rates of horsts and grabens up to centimetres per century; rivers incise into horsts. **b**, Engineering timescale in which natural rivers (upper panel) are engineered (lower panel) and sediment transport is modified, leading to incision rates up to centimetres per year. **c**, Event timescale during which flow conditions change rapidly and erosion and deposition processes develop fast; labels d, e and f along the plot line refer to moments of the processes in panels **d**, **e** and **f**, respectively. **d**, Armoured bed with increasing suspended load from upstream and developed small sand dunes. Underneath the armour, a thin mixture of gravel and sand overlays a thick layer of fine sand. **e**, Increased flow disrupting the armour and forming gravel dunes. **f**, Rapid development of scour hole in fine sands^58,59^.

Heterogeneity of the subsurface stratum and recent uneven widening explain the occurrence of extreme scour in the Common Meuse (Fig. 4). Similar scour holes have been reported in the Mekong^48^, Mississippi^49^, Saskatchewan^50^, Mahakam^51^, Tisza^41^, Petit Rhône^13^, Rhine^52^ and Salzach (tributary to Danube)^53^ rivers, as well as in the Mackenzie^54^ and Rhine–Meuse^22,55^ deltas. In 8 out of 11 reported cases, scouring is linked to human interference or excessive sediment mining. In 8 cases, the scours are linked to subsurface sediment heterogeneity (Extended Data Fig. 7), which results from tectonics and, possibly, glaciation, erosion and sedimentation. In most rivers, the lithology of strata directly beneath the riverbeds remains poorly documented, leaving erosion risks unknown. This underlines the urgent need to characterize the geological structure of riverbeds and embanked floodplains before implementing engineering works.

Efforts to mitigate river floods are increasing^16,56^ through measures such as dyke construction and floodplain widening, often integrated with ecosystem restoration^57^. Examples include projects such as Room for the River^4,11^ in the Netherlands, and similar initiatives in Canada (Room for Nature^5^; Freedom Space for Rivers^6^), the USA (levee setbacks^7^), Belgium^8^ and the UK^9^. Scenarios for socio-economic growth and climate change indicate that without mitigation and adaptation measures, global river flood risks, including population exposure, fatalities, direct damage and welfare losses, can increase substantially^3^. Under a 1.5 °C global warming scenario, these risks can rise by up to 60%, 83%, 240% and 0.29%, respectively^3^, and for 2 °C global warming, these impacts nearly double.

This prospect will inevitably lead to new large-scale river interventions, with subsequent changes in flow conditions. The 2021 Meuse flood cautions for unforeseen geomorphological responses, including those stemming from flood risk mitigation measures in incising rivers in a changing climate. Rapid and uncontrollable erosion and deposition pose threats to the stability of riverbeds, riverbanks, flood levels and ecosystems, and imperil the safety of communities in the proximity of rivers. Comprehensive impact assessments of future river projects need to account for altered discharge regimes due to climate change, carefully consider the implementation sequence of measures, and face the threat of rapid erosion during an event. Our observations suggest that a better understanding of surface and subsurface sediments is crucial for building probabilistic flood risk assessments that account for the transient character of bedforms and channel morphology within a flood event.

## Methods

### Assessment of riverbed erosion and deposition

The Dutch river authority Rijkswaterstaat has conducted multibeam echosounder surveys in the Meuse since 2003. The last survey before the 2021 summer flood was in February 2021. Rijkswaterstaat conducted additional multibeam measurements during the waning stage of the flood on 19 July 2021, which were limited by the river’s rapid water level drop. These measurements were supplemented with data from multibeam surveys by the Meuse Program contractor in the months after the flood, focusing on specific project locations. Together, these datasets cover changes in riverbed elevation over 60% of the Common Meuse. The validated point cloud data exhibit both stochastic and systematic errors of less than 5 cm, and the density is at least 10 points per square metre. The point data were converted to a grid with a horizontal resolution of 1 m. Additionally, Rijkswaterstaat continued riverbed monitoring in the weeks after the flood for the impounded and navigable river sections upstream and downstream of the Common Meuse, resulting in a similar bed level grid.

Using the MATLAB TopoToolbox^60^, version 2.3, we identified 16 scour holes exceeding 3 m in depth (about 40% of the flow depth) from the bed level data. We determined the edge of each scour hole by fitting a sigmoid curve to the bed level in all directions as a function of the distance from the deepest point in the scour hole, selecting the inflection point of that function as the bed level of the edge. For the surrounding riverbed elevation, we used the highest contour line enclosing the scour hole. We calculated various characteristics of each scour hole, including maximum depth, area, volume, orientation, and position relative to bends, as well as changes in floodplain width in the Common Meuse. Additionally, we analysed riverbed changes since the first multibeam measurements in 2003 at the identified scour locations to investigate whether any precursory signs could already have indicated scour initiation.

With the aim to setup a sediment balance at a regional scale for the period of the 2021 flood event, we generated a stream-following grid with eight cells across the channel width for the entire Meuse River in the Netherlands, and 250 m in the streamwise direction. With this approach, the width of the cells varied between 10 and 20 m. We determined the average bed level of the main channel for each 250-m section of river length on the basis of the area-weighted average of the eight cells in the cross-section. We calculated the changes in riverbed elevation between February 2021 and the period directly after the flood (Extended Data Fig. 6a). To distinguish between effects from the flood and from human activities, we subtracted maintenance dredging records of the contractors for February–July 2021 from the volumetric differences obtained from the bed level measurements.

### Assessment of gravel layer thinning

Eroding trends cause thinning of the top gravel layer. The bed level observations show a long-term erosion of 2 cm yr^−1^ for the complete Common Meuse, or 0.6 cm yr^−1^ since 1995. However, in the scour section (km 30–40), the average riverbed level since 1995 was stable or even slightly aggrading^36^. We infer that the gravel layer has thinned as a result of the average surface uplift in this region of 3.5 mm yr^−1^ caused by closure of coal mines^44^. If the riverbed is stable, this surface uplift is an indication of the thinning of the gravel layer in the 26 years since 1995 (26 × 0.0035 ≈ 0.1 m).

### Gravel dune dimensions

Gravel dunes have previously been described^45,61^. We calculated dune dimensions during the flood using the sand dune predictor of ref. ^62^, adopting a water depth *h* of 8 m, a bed slope *I* of 5 × 10^−4^, a median grain diameter *D*_50_ of 0.025 m (based on Extended Data Fig. 1c) and a value of 0.045 for τ^*^_c_, the critical Shields number for entrainment in gravel mixtures^61^. The calculated dimensions agreed well with the observed dune height of 1–2 m and length of 50–90 m (Fig. 3h), despite the application to gravel. This confirms that the high sediment mobility during this flood allows non-dimensional dune predictors, typically used for sand dunes, to also be applicable in gravel-bed conditions^45^.

### Bank erosion

Bank protection has been removed along parts of the Dutch Meuse since 2009 to improve the ecological status of the river. Both unprotected and protected banks eroded during the 2021 flood. We analysed LiDAR data from April 2021 and August 2021 (11–13 August), which attain the same accuracy as the multibeam measurements, to establish distances of bank displacement and volumes of bank erosion. The data density was 16 points per square metre, and data were classified as ground, water or vegetation in compliance with the standards specified in LAS 1.4 (ASPRS Standard LIDAR Point Classes). Using vegetation maps and actual information on crops in the fields, we established that parts of agricultural areas and vegetated floodplains were incorrectly identified as bare ground instead of vegetation, especially for the August data. This issue did not hamper identification of the bank erosion lines.

We drew left and right riverbank lines perpendicular to the flow direction, at 10-m intervals (Extended Data Fig. 8a). Each line covers the morphologically active riverbank section. Using a spatial resolution of 10 cm along the lines, we calculated the representative ground elevation from the LiDAR data. For each point along the line, we calculated the median value of all ground-classified data points within a 1-m-diameter circle. Then, we calculated the height and position of the erosion line following ref. ^63^. Along the cross-section from floodplain to river, the erosion line is defined as the first point beyond which three successive points along the cross-section show a transverse slope smaller than 0.09. The procedure was performed for the April and August datasets. We filtered out unrealistic cross-sections, which occurred when: for one dataset the difference in position or height of the erosion line between neighbouring cross-sections exceeded 10 m; or the erosion seemed negative, suggesting unlikely deposition, which can be ascribed to the growth of crops and other vegetation between April and August.

As measurements showed that bank erosion took place almost completely above the average water level, and in several river reaches water levels were erroneously identified as bed level, we assessed erosion volumes by subtracting the parts of the cross-sections above the average water level from each other (Extended Data Fig. 8b). Rijkswaterstaat provided the average water level based on measurements from recent years and numerical simulations. We assumed the erosion area of each cross-section representative for the bank erosion along 10 m of river length to calculate the eroded volume (Extended Data Fig. 8d–f).

### Floodplain deposition

We measured the thickness of new floodplain deposits at nearly 3,000 locations and collected more than 200 sediment samples during a fieldwork campaign along 225 km of the Dutch Meuse River, in the weeks after the event. We analysed the sand content of deposits at more distal overbank zones, by sampling deposits along ten transverse sections to estimate the background sedimentation rate of this extreme event in distal zones (Extended Data Fig. 9). Elevation changes along the riverbanks (Fig. 2b and Extended Data Fig. 10) and on the floodplains were also mapped from LiDAR measurements in August 2021, and aerial images. Although crops and other vegetation hindered the identification of bed elevation changes, the aerial images provided consistent bed elevation information for inaccessible floodplains and locations not sampled during the fieldwork.

#### Proximal zone

We measured the non-cohesive sand and gravel deposit thicknesses in the proximal zone of riverbanks following a standard protocol. The focus areas encompassed all floodplains along the Dutch Meuse River from the border with Belgium (km 2.48) to the delta (km 247). We selected target areas for field surveys using: maps of deposits after the floods of 1993 and 1995 (ref. ^64^); reports of Rijkswaterstaat; high-resolution satellite images; flow path results of two-dimensional (2D) flow simulations at high discharges with overbank flow. We used similar equipment as in the fieldwork during previous high floods^64^. We measured the thickness of fresh deposits with a plastic hollow pipe of 1 m long and 2 cm in diameter, with a centimetre scale, by pressing it through the fresh deposits until higher resistance, of underlying grass sods, was detected. We validated this method regularly by excavating deposits. We applied a high-resolution GPS positioning system (Altus APS3G) for recording the boundaries of deposits and logged data using the app MapSediment (in Dutch, SedimentInKaart), which was developed for this fieldwork to store location, thickness of deposits, sediment sample number, type of sediment, photos and remarks.

We found sand deposits at 59 of the 89 floodplain sites visited between 16 and 27 August. We measured deposit thickness at intervals of at most 25 m along transects parallel to the river. The average density was 66 measurements per hectare. We collected 2,939 thickness measurements and 201 sand samples.

We combined the point measurements of deposit thickness with the boundary lines of the deposits to generate a 3D representation of the deposits. LiDAR data were collected between 11 and 13 August 2021. As this was almost 1 month after the flood, vegetation had already grown, and classification of the ground seemed difficult sometimes. However, by using high-resolution (10 × 10 cm^2^) aerial photographs taken during the same LiDAR campaign, we identified the non-vegetated regions in most cases, and we used the LiDAR data to validate and supplement the field data. Despite local differences, on a floodplain scale, the deposition volumes from field sampling and LiDAR proved to be either in good agreement, or with differences readily explicable (Extended Data Fig. 10). Several areas were not measured in the field, because they were inaccessible, because they were missed, or because deposits were too thick (>1 m) to be measured accurately. For those areas, we used the LiDAR data from April and August 2021 to assess the deposition volumes. We assessed an overall volume uncertainty of approximately 30% (ref. ^65^), on the basis of estimates of the errors in fieldwork and LiDAR.

#### Distal zone

Between km 34 and 141, we sampled the distal zones of the floodplains in straight, curved, wide and narrow river reaches between 19 July and 5 August 2021. Along ten cross-sections transverse to the flow direction, we measured the thickness of deposits, and collected sediment samples. We collected not only sand deposits but also finer deposits, such as silt and clay. From the river to the dykes or higher grounds along the river, the change in sand content in the samples provided a preliminary and rough estimate of the volume of sand deposits outside the proximal zone (Extended Data Fig. 9), here referred to as ‘distal zone sedimentation’ or ‘background sedimentation’ during the flood. Thickness and sand content of the deposits along the cross-sections appeared to be relatively constant along the 107-km river reach. Average values amounted to 60 m^3^ ha^−1^ deposition and 23% sand content in the zone between 80 and 200 m from the riverbank, and 9 m^3^ ha^−1^ deposition and 8% sand content in the zone more than 200 m away from the riverbank. We translated these approximate, average values to background sedimentation rates of sand per river reach between two barrages, using the calculated areas of inundated floodplain zones (80–200 m and >200 m). Assuming a uniform distribution of the background sedimentation in each river reach, we estimated the background sedimentation for the complete Dutch Meuse, yielding a rough order-of-magnitude estimate.

#### Sediment composition

**Extended Data Fig. 5:**
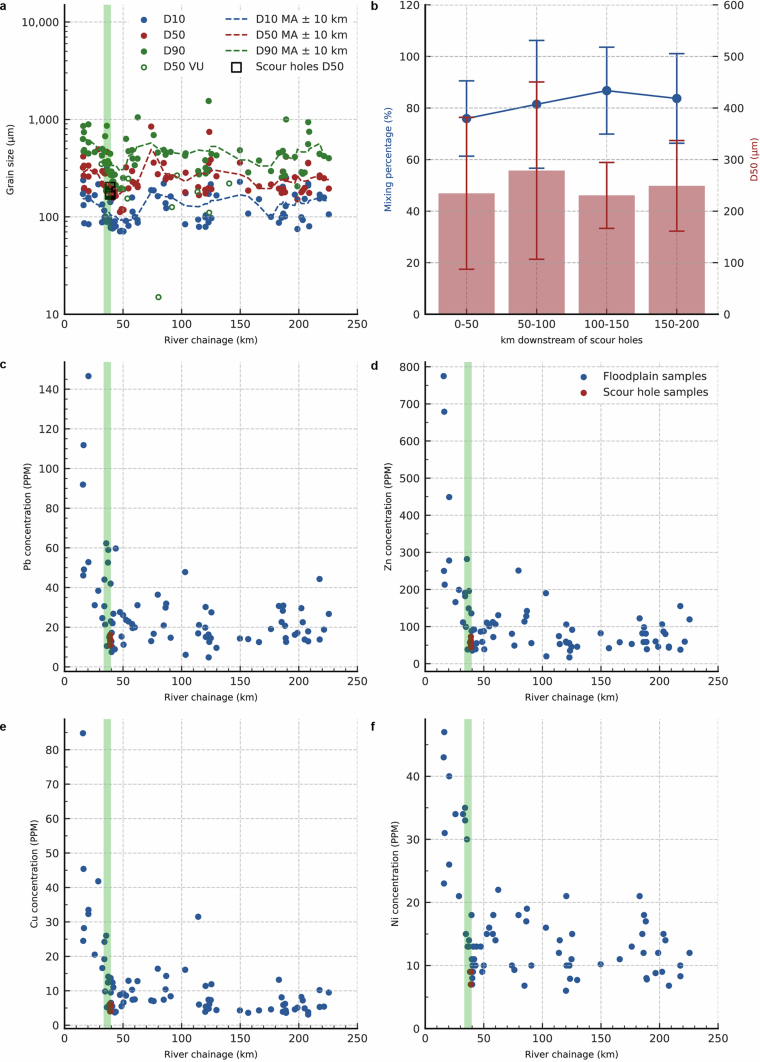
Sand deposit characteristics. **a**, Grain size distribution metrics for proximal zone floodplain deposits (mixed samples, see Methods) with moving average values. *D*_*50*_ values for samples from one scour hole (**□**) and near-river samples (o, “D50 VU” from Extended Data Fig. 9) are added for comparison. In a 20 km section downstream of the scour holes, deposits contain only fine sediment. **b**, Average mixing percentages and average *D*_*50*_ grainsize of floodplain samples, including the standard deviations, for sections of 50 km downstream of the scour holes, showing no significant trend in composition for downstream reaches. **c**-**f**, Concentrations of Pb, Zn, Cu and Ni in sediment samples from XRF analyses. Green bars indicate the scour hole section in the river; scour hole samples were taken at km 39, in the most downstream scour hole. Low concentrations in the scour holes show eroded Neogene sediments (23-5 million B.P.) to be uncontaminated, upstream deposits to be of more recent origin and the Neogene sediments to be deposited on downstream floodplains. Panels b-f are based on Van der Niet^79^.

We dried the 201 sediment samples from the proximal zone campaign, and mixed them for every floodplain with neighbouring samples that showed to be similar, to reduce the laboratory work. We sieved the 87 remaining samples mechanically with a sieve set with mesh diameters of 63, 150, 212, 300, 500, 1,000, 1,410 and 2,000 μm, and determined the combined clay and silt content and characteristic diameters *D*_10_, *D*_50_ and *D*_90_ (Extended Data Fig. 5a). We analysed the samples from the distal zones through laser diffraction^66^, measuring 56 grain size classes between 0.1 and 2,000 μm.

To establish the degree to which fine sediments eroded from the scour holes were deposited on floodplains downstream, we chemically analysed the 87 samples of the proximal zone campaign using X-ray fluorescence spectroscopy^67,68^. For reference, we collected six additional sediment samples from the scour hole at km 39 with a Van Veen grab sampler, and analysed these in the same way as the floodplain samples. These samples represent the Neogene (Miocene) sand that was eroded from the scour holes. From the 26 elements analysed with X-ray fluorescence spectroscopy, 15 elements proved to be significantly present, of which Sr, Ni, Pb, Zn and Cu provided most insights (Extended Data Fig. 5c–f). Through a principal component analysis, identical to previous analyses for the Rhine River^69^, we selected a set of elements best capable of identifying deposits of Miocene sands from the scour holes. We calculated mixing percentages using sample scores of the principal components (Extended Data Fig. 5b). The mixing percentage indicated the fraction of Miocene sands in the deposits downstream of the scour holes.

### Sediment balance analysis

**Extended Data Fig. 6:**
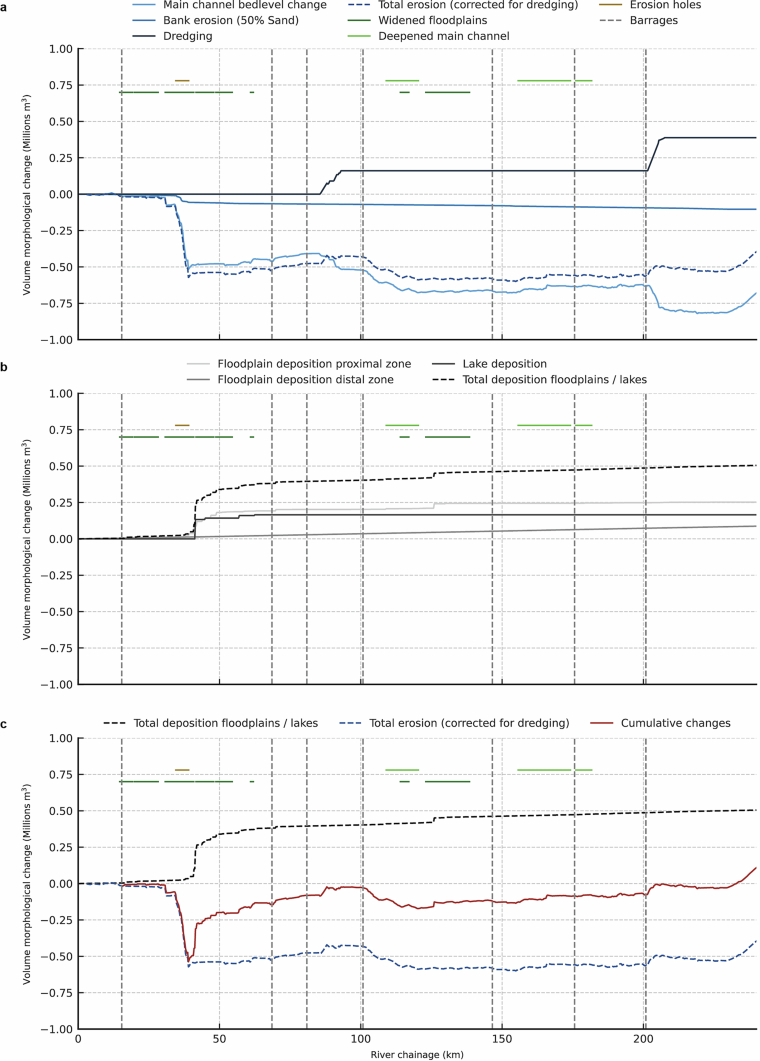
Cumulative Erosion and Deposition Volumes along the Meuse River. **a**, Erosion based on riverbed level information from February and July 2021, LiDAR data from April and August 2021 (for bank erosion), and dredged volumes between February and July 2021 (data Rijkswaterstaat). **b**, Deposition of sand on floodplains (field work and LiDAR) and in several main lakes (multi beam measurements). **c**, Cumulative changes based on information in panels a and b, showing that 50% of the sand eroded from the scour holes was deposited within 5 km downstream of the last scour hole and an additional 30% in the succeeding 25 km.

**Extended Data Fig. 7:**
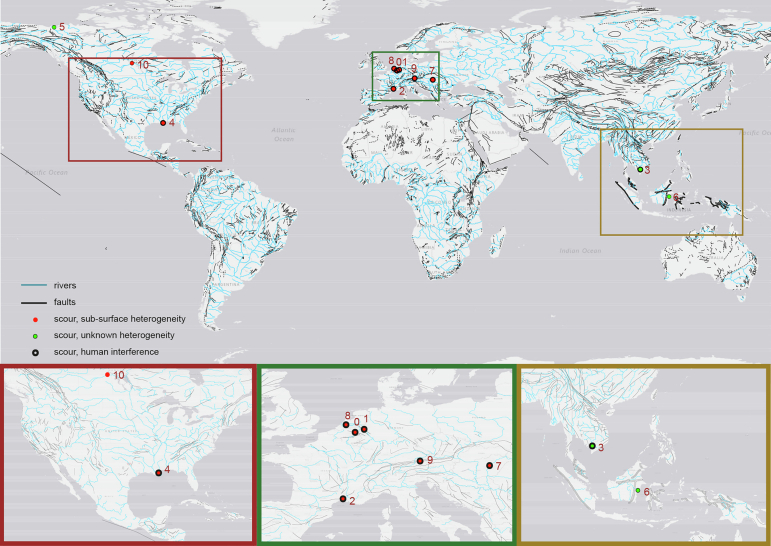
Riverbed scour and tectonic faults. World map with faults^80^ and the 11 cases of riverbed scour referred to in this manuscript (details in bottom panels): 0 – Meuse River, the Netherlands; 1 – Rhine River, Germany; 2 – Petit Rhone, France; 3 – Mekong River, Vietnam; 4 – Lower Mississippi, United States; 5 – Mackenzie Delta, United States; 6 – Mahakam River, Indonesia; 7 – Middle Tisza, Hungary; 8 – Rhine Meuse Delta, the Netherlands; 9 – Salzach River, Austria; 10 – Saskatchewan River, Canada. Markers indicate scour in regions with high subsoil heterogeneity and strong human interference. Many rivers are located in regions with tectonic faults, where irregular sedimentary composition of the subsoil can be expected. The scour cases 0, 1, 6 and 9 correspond to the existence of faults.

**Extended Data Fig. 8:**
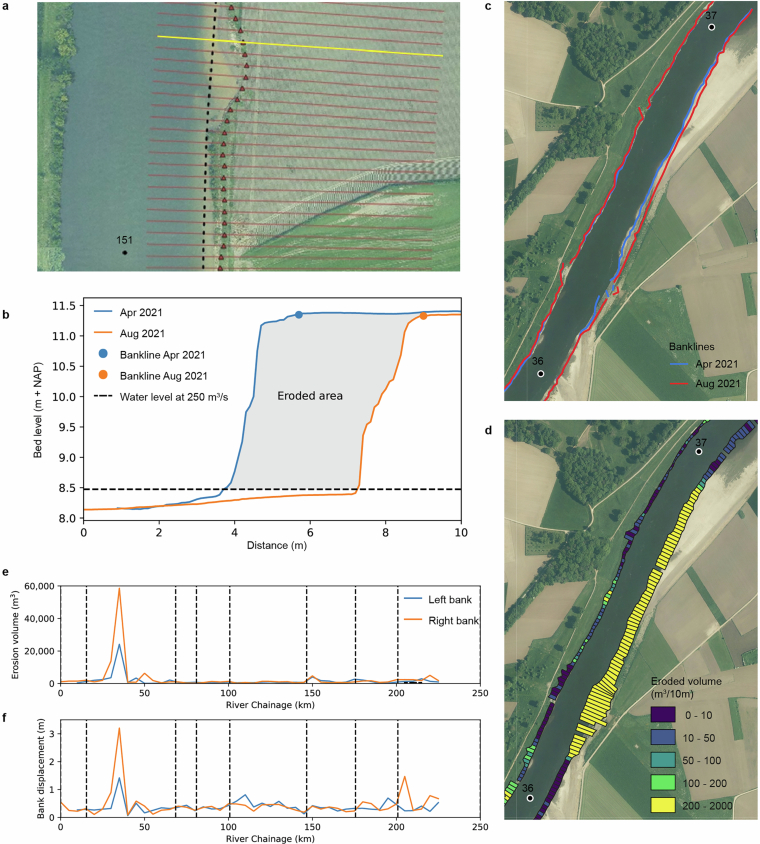
Bank erosion assessment. **a**, Example cross-sections on the right bank near km 151 with estimated erosion line (red triangles). **b**, Cross-section at km 151.14 (yellow line in panel a) and eroded area based on cross-sectional profiles and impoundment level. **c**, Example erosion lines before (blue) and after (red) the flood. **d**, Matching erosion volumes per section of 10 m long (distance in flow direction). **e**, Erosion volume per 5 km river length at the left and right banks. Dotted black lines show the locations of 7 barrages in the river. **f**, Average bank displacement per 5 km river length at the left and right banks.

**Extended Data Fig. 9:**
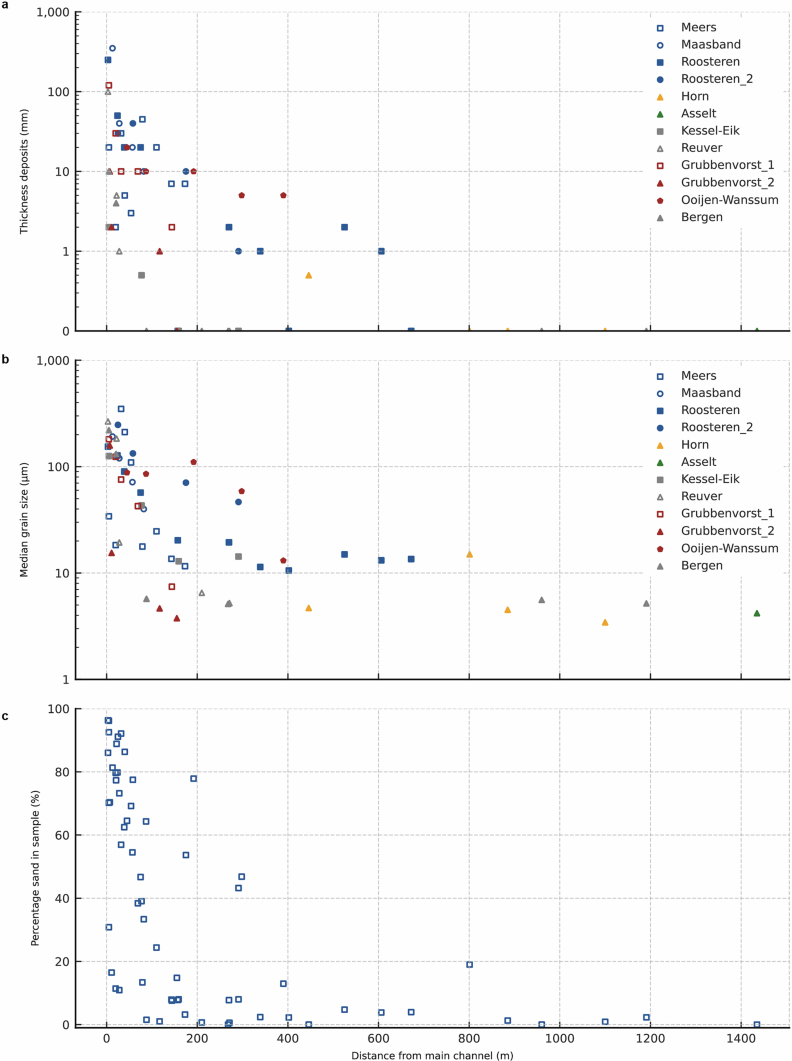
Composition of samples in transverse direction from the main channel at 10 locations. **a**, Thickness of deposits. **b**, Median grain diameter of samples. In panels a and b, locations in the legend are sorted from upstream to downstream. Square, circle and triangle markers indicate respectively inner bends, outer bends, and straight sections. **c**, Sand content in samples.

**Extended Data Fig. 10:**
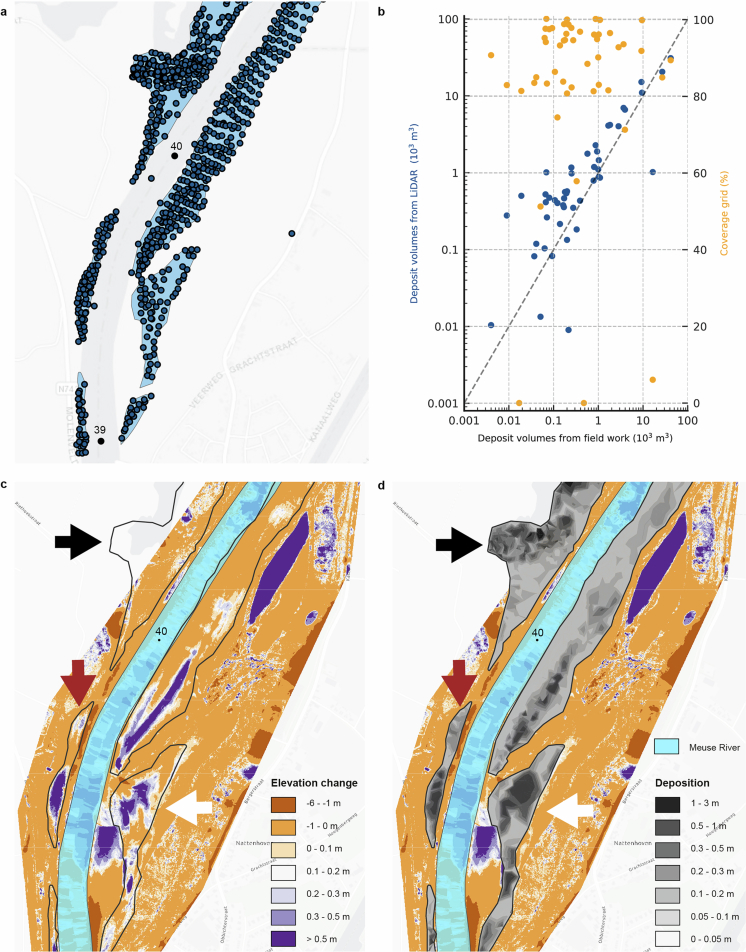
Deposit volumes from field sampling and LiDAR. **a**, Locations of fieldwork thickness measurements and deposition areas (blue). **b**, Deposit volumes from field measurements (x-axis) and LiDAR (primary y-axis) for all Meuse floodplains within the same areas, and relation to coverage of the LiDAR data per area (secondary y-axis). LiDAR-based overestimation can be explained by vegetation or gravel aggradation during the flood prior to sand deposition. LiDAR-based underestimation can be explained by a reduced coverage of LiDAR, i.e. in the large deposition areas of Ooijen-Wanssum and Negenoord (2 largest volumes). Analyses show that the field data are a solid base for the deposition volumes during the flood on the often-vegetated floodplains of the Meuse River where excavation works took place in 2021. LiDAR supplements the field work in areas with thick deposits (> 1 m) and outside survey areas. **c**, Floodplain level changes based on LiDAR data from April 2021 and August 2021. Brown (−1 – 0 m) generally represent small bed level lowering. The dark brown areas at the Eastern edge show overestimation of LiDAR where trees are (incorrectly) classified as ground in April 2021. **d**, Floodplain level changes based on the field surveys, plotted over the LiDAR differences in Panel c. Deposition patterns from LiDAR and fieldwork are identical, despite local differences in deposition depths and volumes. At the red arrow, LiDAR volumes exceed the fieldwork by 25%, probably due to detection of vegetation as ground in August 2021. The white arrow points to the area where LiDAR volumes are 33% lower than estimations from the field surveys, explainable by excavation works in the period April-July 2021, being part of the LiDAR-difference. The black arrow shows an example of underestimation of LiDAR volumes, because LiDAR measurements were missing in April 2021 for this area.

We combined all mean sand erosion and deposition volumes into the cumulative erosion and deposition chart shown in Extended Data Fig. 6.

### Water level and discharge observations

Rijkswaterstaat commissioned a LiDAR survey to measure the water surface near the flood peak. The raw data (density comparable to multibeam measurements, systematic error <3 cm) were converted to a 0.5 × 0.5 m grid. In the section of the scour holes, the LiDAR measurements were taken approximately 15 h after the peak water level. The LiDAR flight was performed in parallel tracks, owing to which the spatial coverage was variable. To construct a water level profile along the central axis from the LiDAR data, the ten grid points closest to each axis point and the MATLAB linear regression function fitlm were used^70^. The water level line was smoothed with a moving average over three data points.

### Numerical modelling of water levels, shear stresses and initial erosion

To assess the impact of discontinuous river widening on cross-section-averaged flow velocity profiles during extreme discharge, we used a calibrated and validated numerical model^71^, which is made using the open-source Delft3D FM Software Suite with flexible mesh. The flexible-mesh approach allows combinations of mesh elements that range from triangles to hexagons. The Delft3D FM model is the successor of the 2D WAQUA model that had been the standard modelling software for the Dutch main rivers for more than 30 years, to simulate flood levels and assess the impact of interventions. The river geometry (Digital Elevation Model in GIS application) was represented on a grid aligned with flow paths as much as possible, with quadrangular cells being preferred over triangles, pentagons or hexagons. Grid cells are 40 m long and main-channel cross-sections contain at least eight cells of at most 20 m wide. The grid cells are up to 40 m wide in the floodplains. The hydraulic roughness in the floodplain sections has been inferred from vegetation maps. The model has been calibrated for low, medium and flood (1995 event) discharge conditions using the data assimilation tool openDA, generating discharge-dependent roughness values for the main river section^71^. For each calibration step, the model schematization was used that best represents the river geometry at the time of the hydrological observations. Measured and simulated water levels for calibration periods during the February 1995 and January 2011 floods deviated less than 5 cm at 31 to 34 stations. The model was validated with data from the high floods of December 1993 and January 1995, showing an average deviation from measured water levels of less than 10 cm at the stations. More details are available in a fact sheet in the 4TU repository (see ‘Data availability’ section below).

Model schematizations of the Dutch Meuse River are available for the geometry of past, present and future situations at https://iplo.nl/thema/water/applicaties-modellen/modelschematisaties/rivieren/. Each geometrical model update is tested on a set of standard boundary conditions to evaluate the geometrical changes. In this study, we compared simulations with the 1995 and 2021 models, which are representative for the geometries before the Meuse Program (dyke strengthening and river widening)^11^ and just before the 2021 flood event, respectively. The discharges and water levels observed during the 2021 flood event were used as boundary conditions for both models. Accordingly, differences between model results can be attributed only to the differences in geometry between 1995 and 2021, which primarily result from uneven river widening of the Meuse Program.

Comparison of simulated water levels and flow velocities reveals the impact of uneven river widening on cross-section average flow velocities in 2021 (Fig. 2c). The simulations also provided shear stresses $\tau =\rho g\frac{{u}^{2}}{{C}^{2}}$, where *ρ* represents the density of water, *g* represents acceleration due to gravity, *u* represents the flow velocity, and *C* is the Chézy roughness coefficient. Many sediment transport predictors relate the excess shear stress above a critical value to the capacity of the flow to mobilize and transport riverbed sediment. The critical shear stress was calculated from *τ*_cr_ = *θ*_cr_ × (*ρ*_s_ − *ρ*) × *g* × *D*_m_, where *θ*_cr_ is the critical Shields value determined using the Shields curve, *ρ*_s_ is the sediment density, and *D*_m_ is the geometric mean grain diameter. The grain sizes used were based on bed load samples collected in 2023 (ref. ^72^). We estimated the geometric mean grain diameter from samples collected from the top layer in five zones that include the centre line, right and left of centre line, and riverbanks left and right. These measured grain sizes were interpolated along 10-km sections.

Comparing the simulated shear stress in 2021 and 1995 to *τ*_cr_ provides a method to assess how river widening affected bed mobility and scour. We find that the transport capacity of the top layer of sediment may have increased up to a factor of 5 in the scoured section of the Common Meuse between 1995 and 2021. Extended Data Fig. 3 suggests that in this reach, the transport capacity during the 2021 event was even larger than for the most extreme flood scenario in 1995.

We also applied the model to calculate initial erosion and deposition rates in the main channel at the peak discharge of the 2021 flood (3,310 m^3^ s^−1^, steady state simulation), which refers to the situation before the feedback with the highly erodible layer began. We use the Meyer-Peter–Müller transport predictor with hiding and exposure^73^ and spatially distributed grain sizes for the top layer from the 2023 monitoring campaign^72^. Although sediment transport was not calibrated, and no bed level changes were simulated, the model run provides initial erosion and deposition rates from shear stress gradients, which are visualized in Extended Data Fig. 3f. Without performing a similar simulation for the 1995 geometry, the initial erosion and deposition rates can be assumed to be much smaller in 1995, as sediment transport gradients causing erosion and deposition scale with flow strength, and were up to a factor of 5 smaller during that year.

We explored whether changes in form drag due to dunes could explain the observed sudden water level rise of up to 0.5 m (ref. ^39^). In an additional simulation with the model, we increased the Manning roughness of the main riverbed in the Common Meuse by 10%, an increase well within the bounds of the roughness increase expected from 1-m-high gravel dunes^74^. This increased flood levels by 15–25 cm in the reach of the scours (km 34.5–39), showing that the dunes are a likely cause of the raised flood water levels.

## Online content

Any methods, additional references, Nature Portfolio reporting summaries, source data, extended data, supplementary information, acknowledgements, peer review information; details of author contributions and competing interests; and statements of data and code availability are available at 10.1038/s41586-025-09305-3.

## Source data

Source Data Fig. 1

Source Data Fig. 2

Source Data Fig. 3

## Figures and Tables

**Extended Data Fig. 4:**
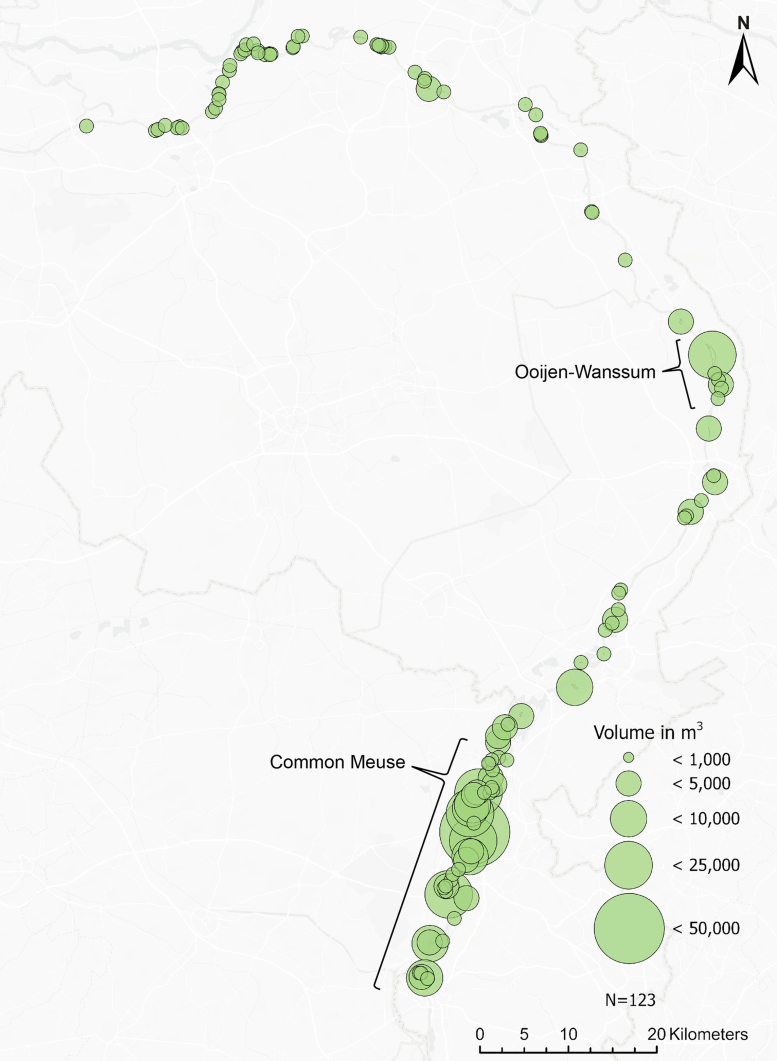
Sand volumes of deposits during the July 2021 flood based on field measurements and LiDAR data. The 89 fieldwork floodplains contained 123 deposition areas. The largest deposits occurred in the Common Meuse and in the Ooijen-Wanssum trajectory, where a large river widening project was completed in 2020. Sand deposits were predominantly found close to the river, particularly on inner bend overbank zones (67%), consistent with typical floodplain deposition patterns^78^.

## Data Availability

All data underlying this study are available via the 4TU repository at 10.4121/462c272c-68ee-4c97-a3be-beb4029c22ed. Source data are provided with this paper.

## Code availability

The Delft3D FM (Flexible Mesh) Software Suite is an open-source hydrodynamic modelling software suite used for simulating water-related processes, including flows, sediment transport, water quality and ecology in 2D and 3D environments. It is the standard modelling software for the Dutch main rivers to simulate flood levels and assess the impact of interventions. The source code is available via Deltares at https://oss.deltares.nl/web/delft3dfm/get-started. Model schematizations of the Dutch rivers can be requested via Informatiepunt Leefomgeving (website in Dutch) at https://iplo.nl/thema/water/applicaties-modellen/modelschematisaties/rivieren/.
